# Efficacy and safety of modular versus monoblock stems in revision total hip arthroplasty: a systematic review and meta-analysis

**DOI:** 10.1186/s10195-023-00731-5

**Published:** 2023-09-16

**Authors:** Daofeng Wang, Hua Li, Wupeng Zhang, Huanyu Li, Cheng Xu, Wanheng Liu, Jiantao Li

**Affiliations:** 1 Senior Department of Orthopedics, The Fourth Medical Center of Chinese PLA General Hospital, Beijing, 100048 China; 2 National Clinical Research Center for Orthopedics, Sports Medicine and Rehabilitation, Beijing, China; 3 School of Medicine, Nankai University, No. 94 Weijin Road, Tianjin, 300071 China; 4 Department of Pharmacology, School of Pharmacy, China Medical University, Shenyang, Liaoning China

**Keywords:** Total hip arthroplasty, Revision, Modular, Monoblock, Tapered fluted stems

## Abstract

**Background:**

Both modular and monoblock tapered fluted titanium (TFT) stems are increasingly being used for revision total hip arthroplasty (rTHA). However, the differences between the two designs in clinical outcomes and complications are not yet clear. Here, we intend to compare the efficacy and safety of modular versus monoblock TFT stems in rTHA.

**Methods:**

PubMed, Embase, Web of Science, and Cochrane Library databases were searched to include studies comparing modular and monoblock implants in rTHA. Data on the survivorship of stems, postoperative hip function, and complications were extracted following inclusion criteria. Inverse variance and Mantel–Haenszel methods in Review Manager (version 5.3 from Cochrane Collaboration) were used to evaluate differences between the two groups.

**Results:**

Ten studies with a total of 2188 hips (1430 modular and 758 monoblock stems) were finally included. The main reason for the revision was aseptic loosening. Paprosky type III was the most common type in both groups. Both stems showed similar re-revision rates (modular vs monoblock: 10.3% vs 9.5%, *P* = 0.80) and Harris Hip Scores (WMD = 0.43, *P* = 0.46) for hip function. The intraoperative fracture rate was 11.6% and 5.0% (*P* = 0.0004) for modular and monoblock stems, respectively. The rate of subsidence > 10 mm was significantly higher in the monoblock group (4.5% vs 1.0%, *P* = 0.003). The application of extended trochanteric osteotomy was more popular in monoblock stems (22.7% vs 17.5%, *P* = 0.003). The incidence of postoperative complications such as periprosthetic femoral fracture and dislocation was similar between both stems.

**Conclusions:**

No significant difference was found between modular and monoblock tapered stems as regards postoperative hip function, re-revision rates, and complications. Severe subsidence was more frequent in monoblock stems while modular ones were at higher risk of intraoperative fracture.

*Level of evidence*: Level III, systematic review of randomized control and non-randomized studies.

*Trial Registration*: We registered our study in the international prospective register of systematic reviews (PROSPERO) (CRD42020213642).

**Supplementary Information:**

The online version contains supplementary material available at 10.1186/s10195-023-00731-5.

## Introduction

With the rapidly increasing number of primary total hip arthroplasties (THAs), there is a concomitant requirement for revision THA (rTHA). Failed THA generally occurs with some extent of femoral, especially proximal, bone defect, limiting the potential of bone ingrowth and rendering adequate fixation of the stem in the revision procedure quite challenging. Different femoral implants have been developed, based on different concepts of modularity and fixation, and have obtained remarkable clinical outcomes and survivorship. The earliest solution was an extensively porous-coated monoblock cylindrical cobalt-chrome stem, which was the gold standard in revision THA in North America for a few decades [1, 2]. This stem, implementing the traditional concept of “scratch fit,” relies on distal fixation at the femoral isthmus and bypasses bone-deficient regions in the metaphysis [3]. Though this stem has provided considerable long-term survivorship (88–96.5% at 10 years) in femoral revision [3–5], there remain concerns regarding the relatively high incidence of intraoperative fracture, thigh pain, and stress shielding of the proximal femur. Additionally, torsional remodeling of the proximal femur after primary THA (usually varus and retroversion) will not allow independent adjustment of femoral anteversion when this stem is used, as the bow restricts the prosthetic position. Proper anteversion may not be achieved. In the setting of severe femoral bone deficiency (Paprosky type IV), due to insufficient isthmic support (4–5 cm), the survival of the stem deteriorated, with a mechanical failure rate of 37.5% [6].

The tapered fluted monoblock titanium stem was developed to mitigate these defects. The stem, which engages a relatively short diaphyseal cortex, achieves both axial and rotational stability through tapered geometry and sharp longitudinal flutes. With a lower modulus of elasticity compared with cobalt-chrome, titanium decreases the modulus mismatch between the stem and the host bone, resulting in less thigh pain and less proximal femoral stress shielding [7, 8]. Owing to the conical body design, adjustment of the stem version can be conducted easily. Previous studies have shown tapered stems could provide superior initial fixation stability compared with cylindrical stems in the scenario of severe bone loss, and present promising clinical results [9, 10]. However, the risk of early stem subsidence and sequent hip instability exists.

A modular design of fluted tapered titanium stem was then developed to counter these concerns and provide greater intraoperative flexibility. In the modular stem procedure, the proximal and distal femur are prepared independently. Immediate stability can be permitted with distal fixation. Meanwhile, optimization of hip biomechanics including offset restoration, leg length correction, and stem version adjustment can be achieved with the proximal body of varying options, intraoperatively. When compared with an extensively porous-coated monoblock cylindrical cobalt-chrome stem, the tapered fluted modular titanium stem yielded improved outcomes [11, 12]. But there are several disadvantages when using modular devices, such as intraoperative fractures, modular junction fatigue fracture, corrosion, and higher implant cost.

Some researchers have compared the differences between modular and monoblock tapered fluted titanium stems and the results are still uncertain. In the study published by Cohn et al. [13], the postoperative Harris Hip Score (HHS) of revision patients in the modular group was 70.7 versus 73.9 in the monoblock group, while Yacovelli et al. reported a postoperative Hip dysfunction and Osteoarthritis Outcome Score for Joint Replacement (HOOS, JR.) of 74.3 in the modular group versus 63.8 in monoblock group, although the two studies showed no statistical significance [14]. Koutalos et al. [15] performed a systematic review to compare the clinical outcomes between the two stems and found that the tapered fluted monoblock titanium stem could provide similar clinical results to the modular stem, but all of the studies involved in their research were with observational cohort design, rather than comparative cohort studies, making the conclusion not rigorous enough.

Therefore, we carried out the present systematic review and meta-analysis to compare the principal complications and clinical outcomes of the two main types of revision hip stems directly after the procedure.

## Material and methods

### Study description

We registered our study in the International Prospective Register of Systematic Reviews (PROSPERO) (CRD42020213642). This work was conducted in line with PRISMA (Preferred Reporting Items for Systematic Reviews and Meta-Analyses) and AMSTAR (Assessing the methodological quality of systematic reviews) Guidelines [16, 17].

### Search strategy and eligibility criteria

PubMed, Embase, Web of Science, and Cochrane Library databases were searched in October 2022. The search terms are listed in Table 1. We developed specific search strategies for each database and references of the identified studies were checked for potential eligibility.

**Table 1:** Search strategy in PubMed

Step	Query	Results
#1	Hip	186,251
#2	Revision	195,545
#3	Modularity OR modulus OR modular	85,162
#4	Nonmodular OR monoblock OR monolithic OR single	1,985,488
#5	#1 AND #2 AND #3 AND #4	217

Query: ("Hip"[MeSH Terms] OR ("Hip"[MeSH Terms] OR "Hip"[All Fields])) AND ("revise"[All Fields] OR "revised"[All Fields] OR "revisers"[All Fields] OR "revises"[All Fields] OR "revising"[All Fields] OR "revision"[All Fields] OR "revisions"[All Fields]) AND ("modular"[All Fields] OR "modularities"[All Fields] OR "modularity"[All Fields] OR "modularization"[All Fields] OR "modularized"[All Fields] OR "modularizing"[All Fields] OR "modulars"[All Fields] OR "modulus"[All Fields] OR ("modular"[All Fields] OR "modularities"[All Fields] OR "modularity"[All Fields] OR "modularization"[All Fields] OR "modularized"[All Fields] OR "modularizing"[All Fields] OR "modulars"[All Fields])) AND ("nonmodular"[All Fields] OR ("monoblock"[All Fields] OR "monoblocks"[All Fields]) OR ("monolith"[All Fields] OR "monolith s"[All Fields] OR "monolithic"[All Fields] OR "monolithically"[All Fields] OR "monolithics"[All Fields] OR "monoliths"[All Fields]) OR ("single person"[MeSH Terms] OR ("single"[All Fields] AND "person"[All Fields]) OR "single person"[All Fields] OR "single"[All Fields] OR "singles"[All Fields]))

**Fig. 1:**
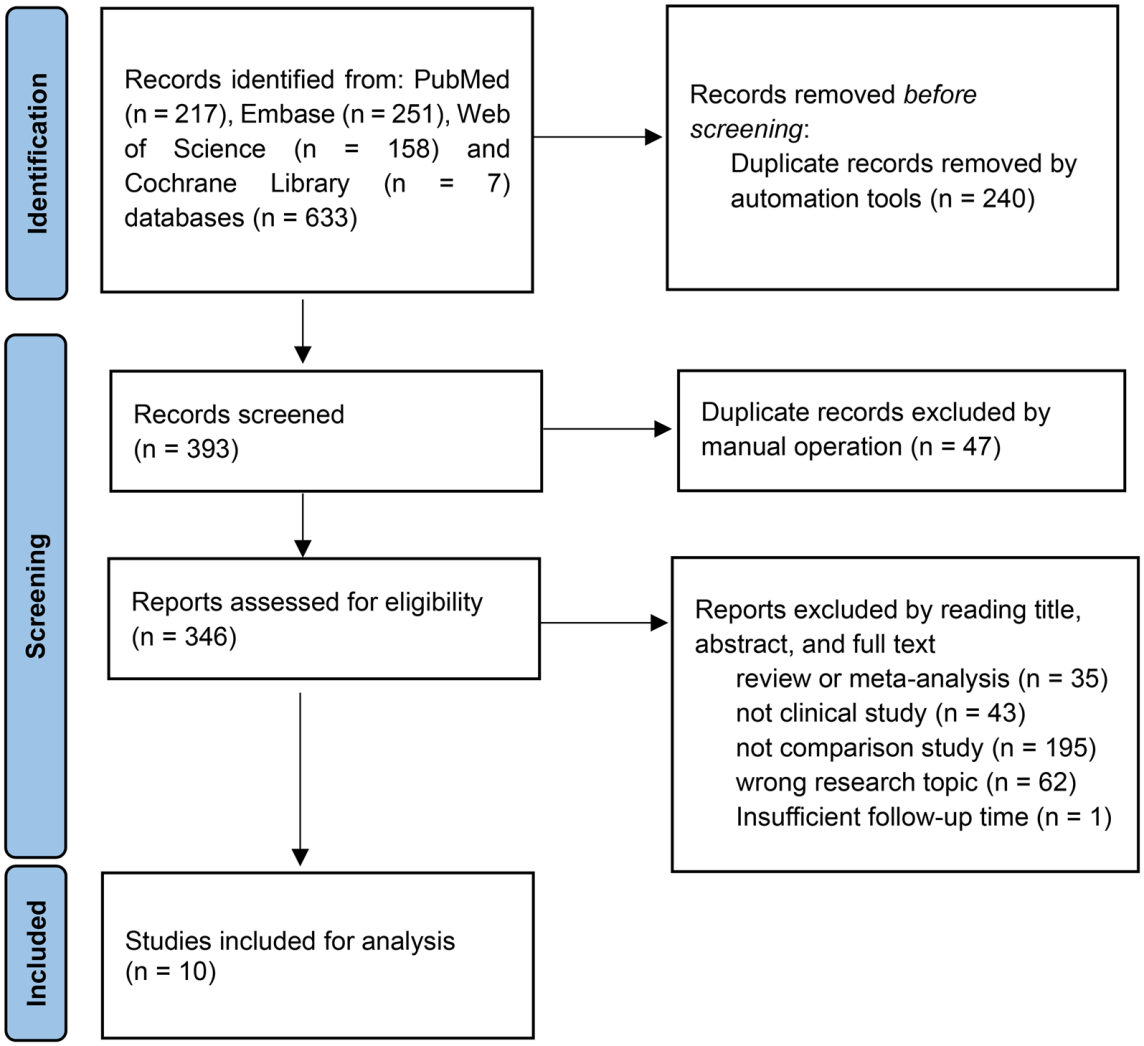
Flow diagram of the selection of studies

The following inclusion criteria were used to identify eligible studies: publications reporting on the outcome of modular and monoblock fluted tapered stems in hip revision surgeries; comparative study design; follow-up duration > 2 years. Furthermore, we excluded non-English language reports, case reports, conference abstracts/posters, or reviews. After the removal of duplicates, two orthopedic surgeons independently reviewed the titles and abstracts to screen for potentially eligible studies. Full texts were then assessed independently by the same two reviewers to identify the final list of publications suitable for inclusion in the current study. If disagreement occurred, a third senior orthopedic surgeon was consulted for final assessment and consensus. The flow diagram for the identification of studies is summarized in Fig. 1.

### Data extraction

After the final list of included studies was set, data were extracted, including information on the publication, patient attributes, and operative and postoperative information. The primary outcomes of interest were the survivorship of stems and the follow-up postoperative hip function (Harris Hip Score, HHS). Intraoperative complications and postoperative complications were extracted as secondary outcomes. If the necessary information could not be extracted from the original paper, we contacted the corresponding author to request additional information.

### Quality assessment

The quality of the included studies was assessed independently by two reviewers. In this regard, the Newcastle–Ottawa Scale (NOS) for cohort studies was used [18]. When disagreement occurred, a third senior orthopedic surgeon was consulted for final consensus.

### Statistical analysis

Review Manager (version 5.3 from Cochrane Collaboration) was used to perform the statistical analysis, with *P* < 0.05 as a threshold of statistical significance. For continuous data with standard deviation, meta-analysis was performed to calculate the weighted mean difference (WMD) with 95% confidence intervals (CIs) using the inverse variance (IV) method. When comparing the incidence of dichotomous data, such as revision or complications, the odds ratio (OR) was calculated using the Mantel–Haenszel (M–H) method. We used the *I*^2^ statistic and *Q* test to measure heterogeneity. If *I*^2^ < 50% and the *p*-value for the *Q* test > 0.05, the studies were interpreted as minimally heterogeneous, and a fixed-effects model was applied for the meta-analysis. A random-effects model was applied when *I*^2^ > 50% or the *p*-value for the *Q* test was < 0.05, which indicated that the data were of high heterogeneity. Other results were presented as a descriptive summary.

## Results

### Overview of search results

There were 633 studies identified in the initial search. After excluding duplications and non-English publications, 346 studies were further assessed by the titles, abstracts, and full-text review for eligibility. As a result, 10 studies were included in the final analysis (Fig. 1). All of these studies were retrospective cohort designs. A total of 1430 hips with modular stems (modular group) and 758 hips with monoblock stems (monoblock group) were identified. Paprosky type III was the most common type of femoral bone defect in the modular group (62%, 600/966) and monoblock group (59%, 375/631), respectively. The main reason for the revision was aseptic loosening. The average age of patients ranged from 78 to 87.4 years old. The characteristics of the patients in the two groups are summarized in Table 2. Patients in all 10 studies were followed up for more than 2 years (2.5 to 8.5 years). In each study, the duration of follow-up was comparable between the two groups (Table 2). The primary and secondary outcomes of included studies are shown in Table 3.

**Table 2:** Characteristics of the included articles

Article	Stem	Patients	Age	Paprosky classification	Follow-up (years)	Reasons for revision
Feng [19]	Modular	108	69.1 ± 7.5	18I 54II 24IIIA 12IIIB	8.5	96L 6I 6D
	Monoblock	110	67.6 ± 7.9	20I 60II 25IIIA 5IIIB	8.5	95L 5I 6D
Huang [20]	Modular	139	61.2 ± 10.9	2I 12II 65IIIA 47IIIB 13IV	6.3	119L 16I 4PFF
	Monoblock	114	59.8 ± 13.2	1I 12II 60IIIA 34IIIB 7IV	5.1	96L 13I 5PFF
Cohn [13]	Modular	67	67.2 ± 13.0	11I 14II 26IIIA 9IIIB 5IV	6.3	33L 17I 12PFF 5O
	Monoblock	78	60.2 ± 12.1	2I 25II 41IIIA 5IIIB	4.1	26L 34I 12PFF 3O
Yacovelli [14]	Modular	225	65.6 ± 12.6	57II 105IIIA 45IIIB 11IV	3.5	95L 44I 61PFF 5D 20O
	Monoblock	63	62.6 ± 14.2	6I 20II 24IIIA 10IIIB 3IV	2.4	6L 26I 8PFF 23O
Chair [21]	Modular	103	NA	30I 44II 17IIIA 12IIIB	2.5	NA
	Monoblock	43	NA	5I 19II 12IIIA 7IIIB	2.5	NA
Chair [22]	Modular	106	NA	30I 45II 18IIIA 13IIIB	3.0	NA
	Monoblock	80	NA	3I 28II 34IIIA 11IIIB 4IV	3.0	NA
Huang [23]	Modular	160	61.8 ± 10.7	2I 13II 75IIIA 55IIIB 15IV	6.3	139L 17I 4PFF
	Monoblock	129	60.2 ± 12.9	1I 12II 66IIIA 41IIIB 9IV	5.0	111L 13I 5PFF
Moreta [24]	Modular	24	78.3 ± 7.1	NA	5.0	24PFF
	Monoblock	19	75.7 ± 6.9	NA	5.0	NA
Zeng [25]	Modular	73	62.5	73 III IV	3.9	41L 15I 9PFF 8D
	Monoblock	19	NA	19 I II	3.9	11L 4I 4D
Chatziagorou [26]	Modular	425	77.2	NA	3.6	425PFF
	Monoblock	103	77.2	NA	4.9	NA

*A* aseptic loosening, *L* aseptic loosening, *I* infection, *PFF* periprosthetic femoral fracture, *D* dislocation, *O* other reasons such as instability, local discomfort, *NA* not available

**Table 3:** Primary and secondary outcomes of included studies

Article	Stem	Pre-HHS	Post-HHS	Subsidence (mm)	Intraoperative fracture	Dislocation	PFF	Aseptic loosening	Infection	Reoperation
Feng [19]	Modular	40.5 ± 6.1	86.4 ± 3.9	0.92	18/108	0/108	2/108	4/108	1/108	5/108
	Monoblock	40.1 ± 6.6	85.5 ± 3.8	2.2	5/110	3/110	2/110	5/110	0/110	5/110
Huang [20]	Modular	39.5 ± 13.2	86.1 ± 8.1	NA	NA	NA	NA	NA	NA	NA
	Monoblock	41.4 ± 14.0	86.2 ± 10.24	NA	NA	NA	NA	NA	NA	NA
Cohn [13]	Modular	46.2 ± 21.9	70.7 ± 17.9	2.17	6/67	8/67	2/67	8/67	7/67	15/67
	Monoblock	42.7 ± 18.5	73.9 ± 19.7	3.13	3/78	4/78	1/78	8/78	8/78	14/78
Yacovelli [14]	Modular	52.7 ± 18.8	74.3 ± 22.9	3.55	NA	2/225	1/225	10/225	3/225	13/225
	Monoblock	54.7 ± 15.1	63.8 ± 26.0	2.44	NA	0/63	1/63	2/63	4/63	6/63
Chair [21]	Modular	NA	NA	NA	0/103	6/103	5/103	1/103	7/103	4/103
	Monoblock	NA	NA	NA	1/43	3/43	0/43	5/43	7/43	7/43
Chair [22]	Modular	NA	NA	3.9	NA	NA	NA	NA	NA	NA
	Monoblock	NA	NA	2.3	NA	NA	NA	NA	NA	NA
Huang [23]	Modular	40.1 ± 13.8	85.2 ± 9.8	1.0	27/160	3/160	2/160	5/160	1/160	6/160
	Monoblock	41.8 ± 13.8	86.1 ± 10.1	1.9	9/129	0/129	2/129	1/129	3/129	4/129
Moreta [24]	Modular	NA	73.6 ± 12.6	1.75	NA	4/24	NA	NA	NA	NA
	Monoblock	NA	73.1 ± 10.2	1.0	NA	3/19	NA	NA	NA	NA
Zeng [25]	Modular	NA	NA	NA	NA	NA	NA	NA	NA	NA
	Monoblock	NA	NA	NA	NA	NA	NA	NA	NA	NA
Chatziagorou [26]	Modular	NA	NA	NA	NA	23/425	NA	53/425	16/425	69/425
	Monoblock	NA	NA	NA	NA	7/103	NA	13/103	69/103	14/103

*HHS* Harris Hip Score, *PFF* periprosthetic femoral fracture, *NA* not available

### Risk of bias in studies

Designs of all the included studies were cohort studies and most of them had excellent selection quality of patients, good comparability between groups, and reasonable assessment of outcomes, as shown in Additional file 1: Table S1.

### Re-revisions

**Fig. 2:**
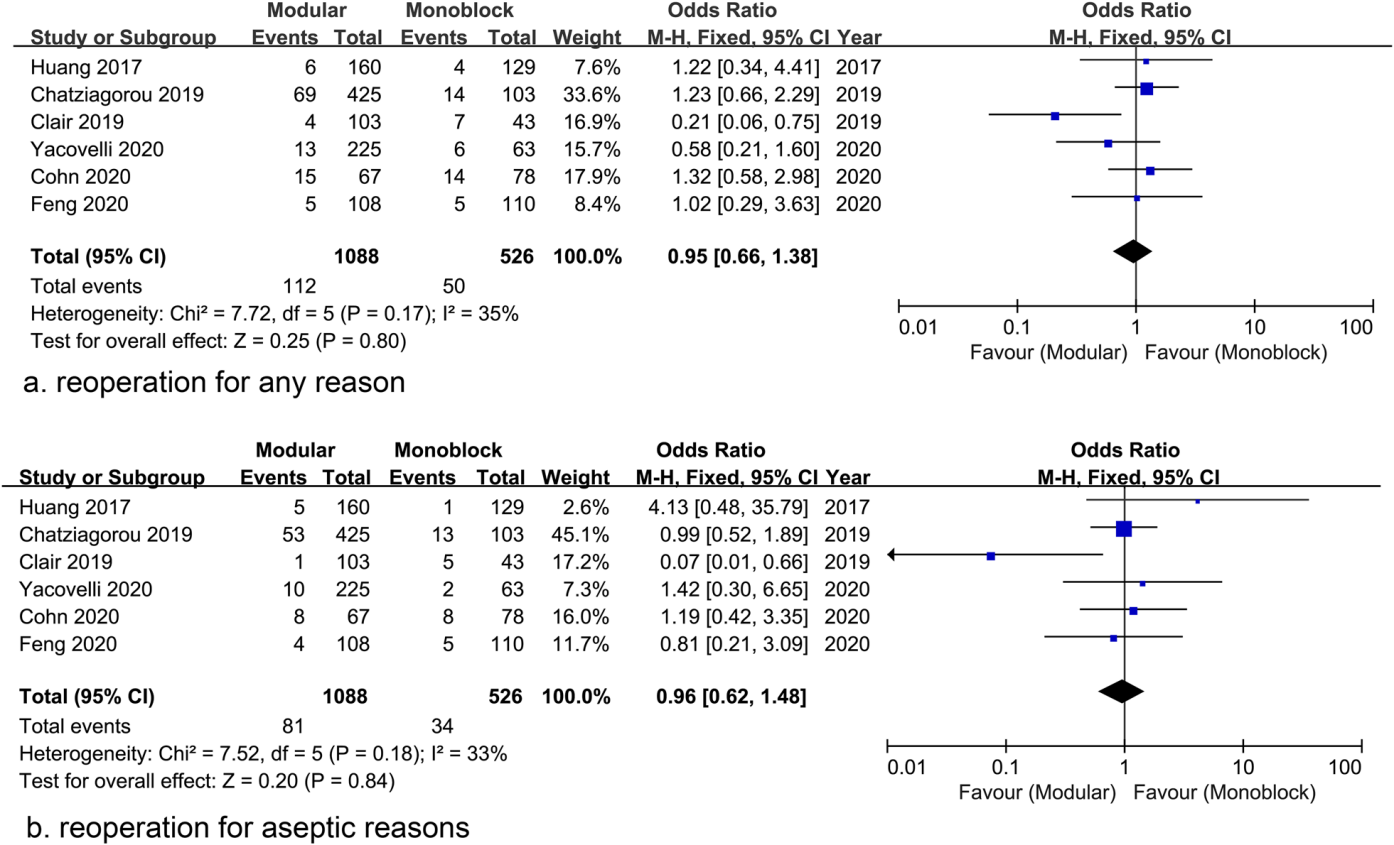
Forest plots of reoperation for any reason (**a**) and aseptic reason (**b**) indicating no significant difference between stems

The re-revision rate for any reason was reported in 6 studies and the pooled data showed no statistical difference between the two groups [Modular group: 112/1088 (10.3%) vs Monoblock group: 50/526 (9.5%); OR = 0.95; 95% CI 0.66 to 1.38; *P* = 0.80; Heterogeneity: *I*^2^ = 35%, *P* = 0.17] (Fig. 2a). The pooled re-revision rate for aseptic reasons was also comparable between the two groups [Modular group: 81/1088 (7.4%) vs Monoblock group: 34/526 (6.5%); OR = 0.96; 95% CI 0.62 to 1.48; *P* = 0.84; Heterogeneity: *I*^2^ = 32%, *P* = 0.20] (Fig. 2b).

### Postoperative hip function

**Fig. 3:**
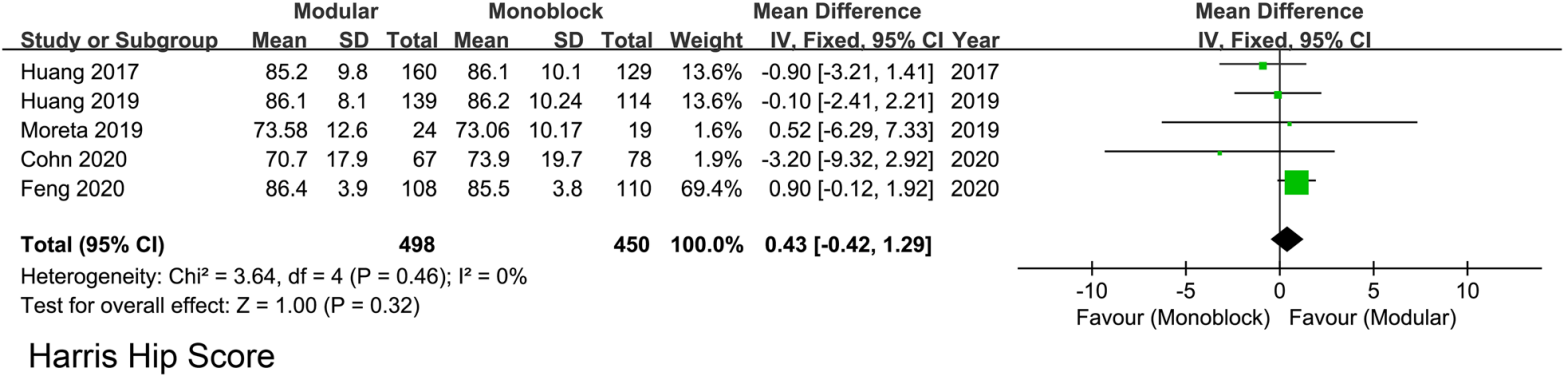
Forest plots of Harris Hip Score indicating no significant difference between stems

Seven studies reported postoperative hip function estimation and data from 5 studies could be further pool-analyzed with the scale of HHS. The postoperative HHS in the modular and monoblock groups ranged from 70.7 to 86.4 points (weighted mean: 85.77), and from 73.1 to 86.2 points (weighted mean: 85.34), respectively. The difference between the two groups was insignificant (WMD = 0.43; 95% CI − 0.42 to 1.29; *P* = 0.32; Heterogeneity: *I*^2^ = 0%; *P* = 0.46) **(**Fig. 3**)**.

### Construction strategy

**Fig. 4:**
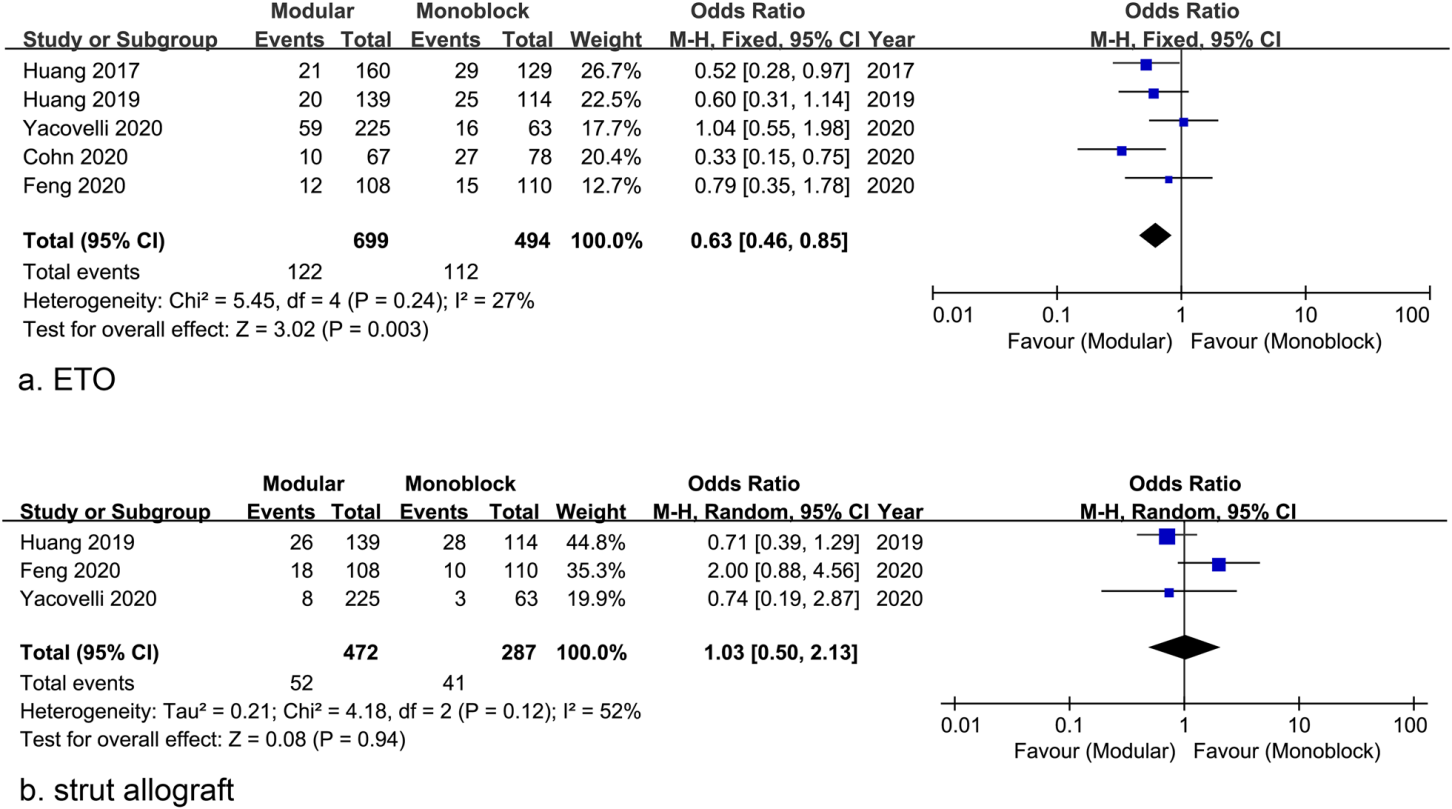
Forest plots of construction strategy demonstrating that the ETO was more frequently used in the monoblock group (**a**) but there was no significant difference in strut allograft between stems (**b**)

Five studies reported the intraoperative application of extended trochanteric osteotomy (ETO) and the pooled analysis showed that ETO was more frequently utilized in the monoblock group [122/699 (17.5%) vs 112/494 (22.7%); OR = 0.63; 95% CI 0.46 to 0.85; *P* = 0.003; Heterogeneity: *I*^2^ = 27%, *P* = 0.24] **(**Fig. 4a**)**.

Three studies reported the intraoperative application of strut allograft and the pooled analysis showed no difference [Modular group: 52/472 (11.0%) vs Monoblock group: 41/287 (14.3%); OR = 1.03; 95% CI 0.50 to 2.13; *P* = 0.94; Heterogeneity: *I*^2^ = 52%, *P* = 0.12] **(**Fig. 4b).

### Complications

**Fig. 5:**
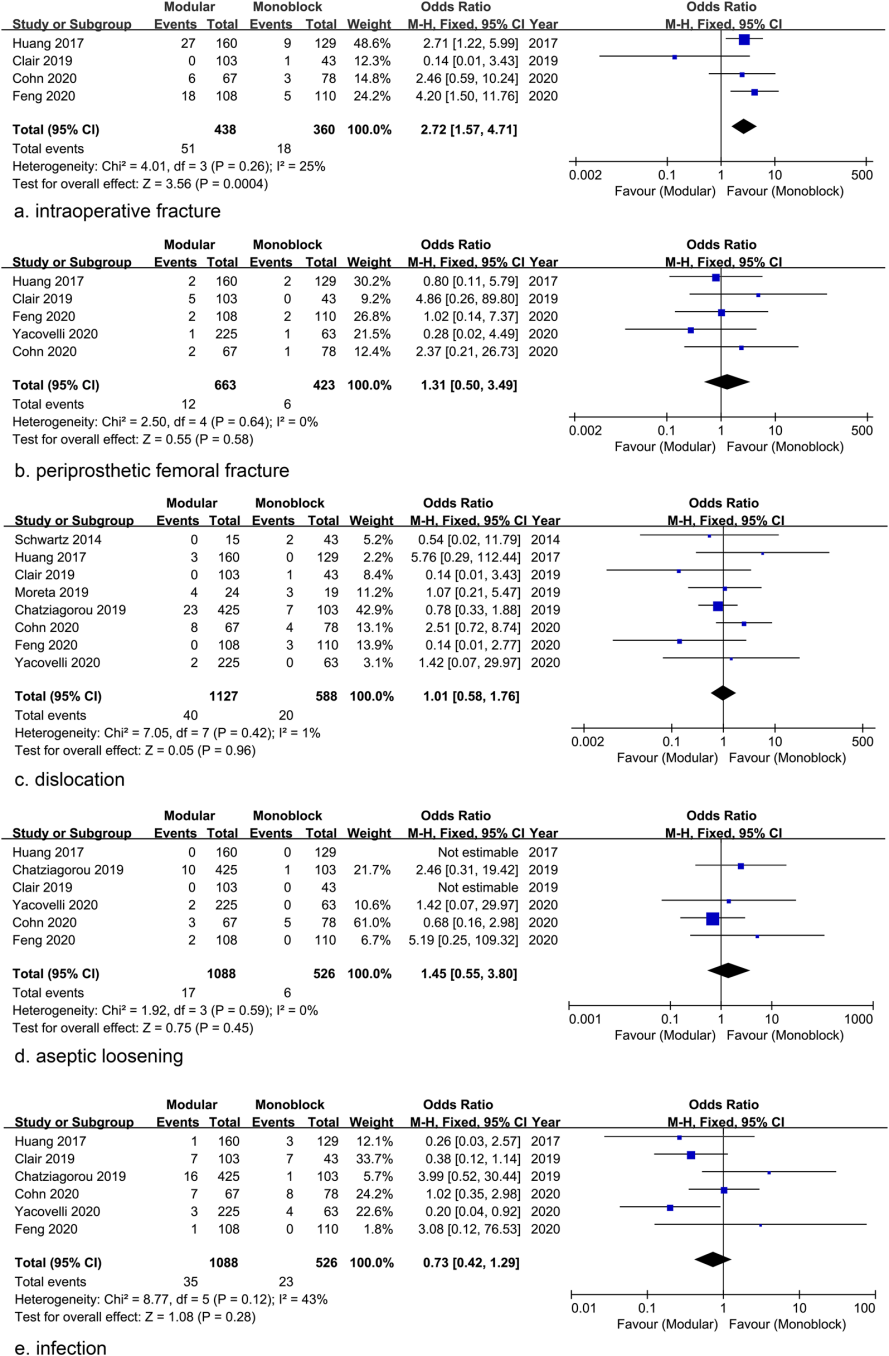
Forest plots of complications showing the higher incidence of intraoperative fracture in modular stems (**a**), but no significant difference in periprosthetic femoral fracture (**b**), dislocation (**c**), aseptic loosening (**d**), or infection (**e**), between stems

Intraoperative fracture data were reported in 4 studies and the pooled analysis showed the modular group had a higher incidence [51/438 (11.6%) vs 18/360 (5.0%); OR = 2.72; 95% CI 1.57 to 4.71; *P* = 0.0004] **(**Fig. 5a**)**. Five studies reported the incidence of postoperative periprosthetic femoral fracture and the pooled estimation reflected no statistical difference [Modular group: 12/663 (1.8%) vs Monoblcok group: 5/423 (1.2%); OR = 1.31; 95% CI 0.50 to 3.49; *P* = 0.58] **(**Fig. 5b**)**. The incidence of dislocation was reported in 8 studies and the pooled analysis also showed no difference [Modular group: 40/1127 (3.5%) vs Monoblock group: 20/588 (3.4%); OR = 1.01; 95% CI 0.58 to 1.76; *P* = 0.96] **(**Fig. 5c**)**. Six studies reported the incidence of aseptic loosening [Modular group: 17/1088 (1.6%) vs Monoblock group: 6/526 (1.1%); OR = 1.45; 95% CI 0.55 to 3.80; *P* = 0.45] **(**Fig. 5d**)** and infections [Modular group: 35/1088 (3.2%) vs Monoblock group: 23/526 (4.4%); OR = 0.73; 95% CI 0.42 to 1.29; *P* = 0.28], none of which showed significant difference through pooled analysis **(**Fig. 5e**)**.

### Subsidence

**Fig. 6:**
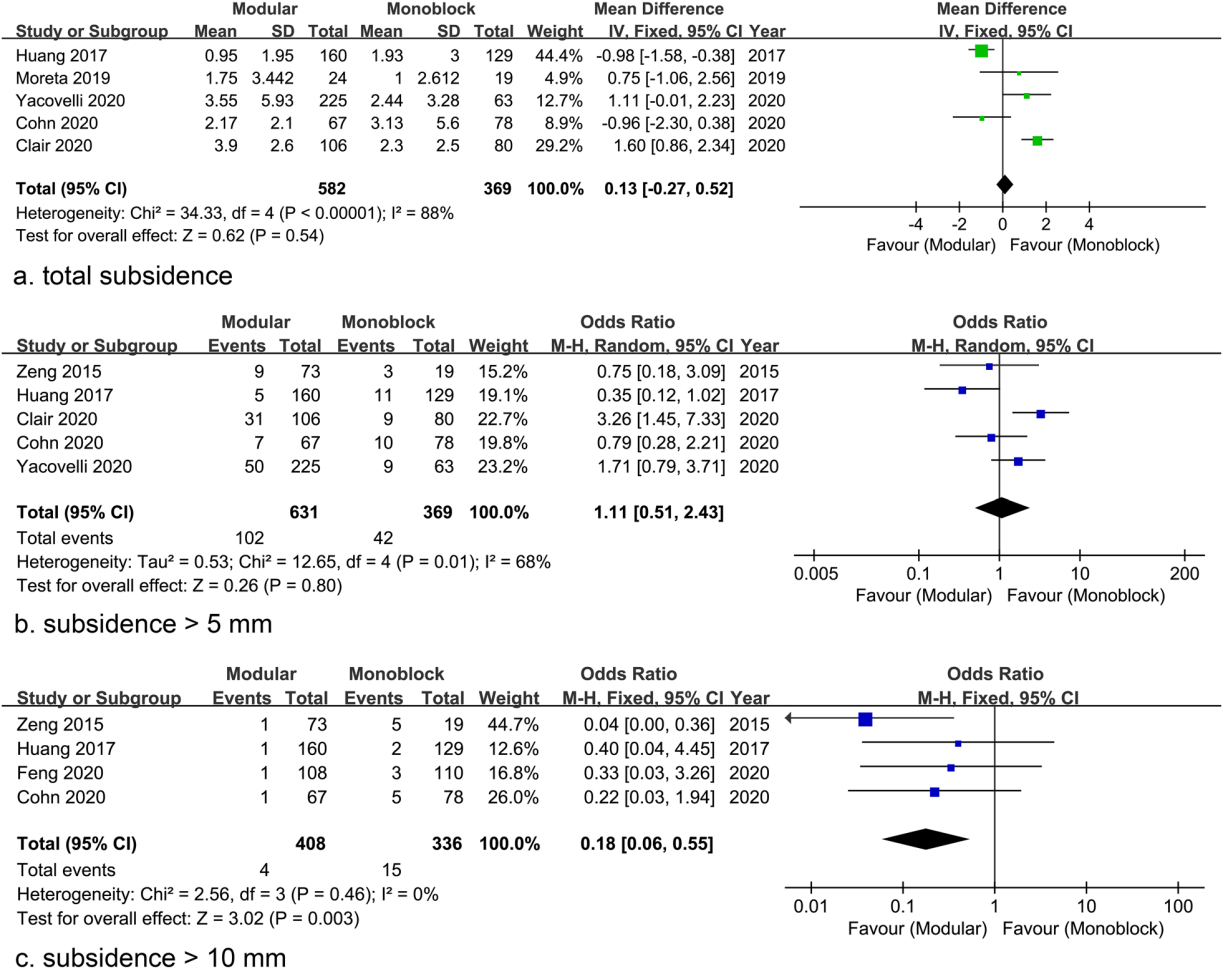
Forest plots of subsidence showing similar rates of total subsidence (**a**) and subsidence > 5 mm (**b**) but a significant difference in the rate of subsidence > 10 mm (**c**), between stems

Five studies reported subsidence data and the pooled analysis showed comparable results with high heterogeneity (WMD = 0.13 mm; 95% CI − 0.27 to 0.52 mm; *P* = 0.54; Heterogeneity: *I*^2^ = 88%; *P* < 0.00001) **(**Fig. 6a**)**. The rate of subsidence > 5 mm was also similar between the two groups [Modular group: 102/631 (16.2%) vs Monoblock group: 42/369 (11.4%); OR = 1.11; 95% = CI 0.51 to 2.43; *P* = 0.80; Heterogeneity: *I*^2^ = 68%, *P* = 0.01] through a pooled estimation of 5 studies **(**Fig. 6b**)**. However, the rate of subsidence > 10 mm was significantly higher in the monoblock group [4/408 (1.0%) vs 15/336 (4.5%); OR = 0.18; 95% CI 0.06 to 0.55; *P* = 0.003; Heterogeneity: *I*^2^ = 0%, *P* = 0.46], based on the available data from 4 studies **(**Fig. 6c**)**.

## Discussion

The tapered fluted titanium (TFT) stem was valued in diverse common options for femoral component revisions and further studied due to prominent axial and rotational stability, ability to improve bone regeneration [27–29], and lower incidence of thigh pain. Monoblock TFT stems have shown promising clinical outcomes but a relatively high incidence of subsidence and dislocation. Modular TFT stems allow distal fixation of the stems for the restoration of proximal hip biomechanics. However, there remain concerns regarding the catastrophic complications associated with the fracture of junctions in modular stems. So far, several influential clinical studies have reported comparisons in clinical outcomes between these two stems. Nevertheless, published outcomes can be controversial, and whether modular or monoblock TFT stems perform better in rTHA is still a subject of interest and debate. To solve this controversy, we conducted this systematic review and meta-analysis to determine which stems would achieve fewer complications and better clinical outcomes.

In general, this analysis indicated that both modular and monoblock tapered stems revealed acceptable and comparable clinical outcomes. There was no significant difference in re-revision and complication risks between groups. Severe subsidence was more frequent in monoblock stems while modular ones were at higher risk of intraoperative fracture. This systematic review was based on cohort studies that directly compared the long-term clinical outcomes (> 2 years) of both modular and monoblock tapered stems in rTHA, and thus the evidence is of high quality.

Postoperative hip function and survivorship were similar between the modular and nonmodular groups, revealing that both of them can achieve satisfactory results for revision. However, more intraoperative fractures were detected in the modular group. Previous studies have demonstrated that the incidence of intraoperative fracture can reach 16 to 32% in modular stems [19]. A systematic review by Koutalos et al. also reflected that modular stems were associated with a higher risk of intraoperative fracture (7.6% vs 9.2%), albeit based on data from case series studies [15]. A possible reason for this result may be that the modular stems might be more popular when there is a larger bone defect, which is more vulnerable to fracture. Several researchers have recommended the prophylactic use of cerclage to prevent intraoperative fracture [30–33]. Thus, surgeons should be aware of our accumulated evidence and use the modular stems with caution.

This study also found that ETO was more frequently used in the monoblock stems. Adequate exposure of the acetabulum and femur and removal of well-fixed femoral components are important in the correction of bony deformity and mechanical stability in rTHA [34]. Osteotomy of the greater trochanter is a common procedure for extensive exposure in this setting [35]. In addition, in cases with proximal femoral varus remodeling or excessive bow, ETO can ensure straight reaming and facilitate stem placement, which is usually difficult for monoblock stems. As the modularity of the proximal and distal parts of stems enables the fixation separately, it may also explain why ETO is less frequently used in modular stems. Since the ETO procedure can facilitate surgical exposure, it may also protect the bone from intraoperative fracture. Previous studies have reported that the use of ETO could reduce the risk of intraoperative fractures and perforation [34–36], which may be associated with a lower risk of intraoperative events in the monoblock groups, as we found in this systematic review and meta-analysis. Nevertheless, it should be noted that the risk of nonunion of ETO is reported to be as high as 15.4% [37]. Ladurner et al. believed that the nonunion of the ETO site could lead to poor osseous support, resulting in inadequate fatigue strength at the junction of the revision stem [38].

Stem subsidence can be of great importance in the clinical setting, and this concern is usually related to the use of TFT monoblock stems. Previous studies reported the rate of subsidence > 10 mm was 15–20% in rTHA with monoblock stems and most of these events occur within the first 3 months [27, 39, 40]. Especially when using the first-generation TFT monoblock stems (Wagner SL; Zimmer, Warsaw, IN), the rate of severe subsidence can reach 20% [28, 29, 41, 42]. Though TFT monoblock stems may gain secondary osteointegration and stability after subsidence, high subsidence will jeopardize hip biomechanics and lead to hip instability, leg length discrepancy, and aseptic loosening. A high heterogeneity in the pooled analysis of stem subsidence was detected in our analysis. We further conducted a subgroup analysis according to the degree of subsidence and found that a rate of subsidence > 10 mm was significantly lower in the modular group [4/408 (1.0%) vs 15/336 (4.5%); OR = 0.18; *P* = 0.003], which confirmed this design clinically. Currently, Sandiford et al. have reported the subsidence of the third-generation TFT monoblock stems at a mean of 2 mm [43], which indicates that the modification of stem design helps in decreasing subsidence. When using modern stems, severe subsidence may be blamed on the surgeon experience, surgical technique, bone defect severity, and under-sizing of the component [29, 44, 45]. For the clinical protocol, the modular design of TFT stems is committed to seating the stem at an appropriate depth that can restore leg length and femoral offset, and reduce subsidence with the help of modular components [46].

This study has several limitations. First, though the design of modular and monoblock stems were restricted to TFT stems, the manufacturers varied among different studies, and the bone defect also differed among studies. We failed to complete subgroup analysis due to the paucity of studies. Though mild heterogeneity of primary outcomes was observed, bias still exists. In addition, the included studies were all retrospective studies, which compromised the level of evidence for this systematic review and meta-analysis. Second, the search methodology contained bias due to the possibly unavoidable missing of relevant studies. However, we searched four main databases to identify all the comparative studies between modular and monoblock stems in rTHA. Based on the given available data, we can answer the main questions.

## Conclusion

The current systematic review and meta-analysis did not detect significant differences between modular and monoblock tapered stems as regards postoperative hip function, re-revision rates, or adverse events. Severe subsidence was more frequent in monoblock stems while modular ones were at higher risk of intraoperative fracture. Therefore, more high-quality clinical studies and clinical trials with larger sample sizes are still needed to provide more solid comparison data and conclusions.

### Supplementary Information

**Additional file 1: Table S1. **Newcastle–Ottawa Quality Assessment Scale (NOS) of included studies.

## Data Availability

Data yielded in our study will be made available by the authors to any qualified researchers.
