# Supplementary material for: Efficacy and safety of modular versus monoblock stems in revision total hip arthroplasty: a systematic review and meta-analysis
Source: J Orthop Traumatol. 2023 Sep 16;24:50. doi: 10.1186/s10195-023-00731-5 (PMC10505121; doi:10.1186/s10195-023-00731-5)
Supplement: Supplementary file 1 — Additional file 1: Table S1. Newcastle–Ottawa Quality Assessment Scale (NOS) of included studies. [file 10195_2023_731_MOESM1_ESM.docx]

**Table S1** Newcastle - Ottawa Quality Assessment Scale (NOS) of included studies.

| Author, Year | Study Design | NOS | | |
| --- | --- | --- | --- | --- |
|  |  | Selection | Comparability | Outcome |
| Feng, S 2020 | Cohort study | **☆☆☆** | **☆** | **☆☆☆** |
| Huang, Y 2019 | Cohort study | **☆☆☆** | **☆** | **☆☆** |
| Cohn, MR 2020 | Cohort study | **☆☆☆** | **☆** | **☆☆☆** |
| Yacovelli, S. 2020 | Cohort study | **☆☆☆** | **☆** | **☆☆☆** |
| Clair, AJ 2019 | Cohort study | **☆☆☆** | **☆** | **☆☆** |
| Clair, AJ 2020 | Cohort study | **☆☆☆** | **☆** | **☆☆** |
| Huang, Y 2017 | Cohort study | **☆☆☆** | **☆** | **☆☆☆** |
| Moreta, J 2019 | Cohort study | **☆☆** |  | **☆☆** |
| Zeng, M 2015 | Cohort study | **☆☆☆** | **☆** | **☆☆** |
| Chatziagorou, G 2019 | Cohort study | **☆☆** |  | **☆☆** |
